# A Case of Thoracic Spondylosis Deformans and Multilevel Instrumented Spinal Fusion in an 84-Year-Old Male

**DOI:** 10.1155/2020/8435816

**Published:** 2020-07-03

**Authors:** Amy H. Amabile, J. Raymond Shea, Vishal Desai, Lisa T. Hoglund, Jamie N. Elcock, Anthony Lombardo, Matthew C. Schiffino

**Affiliations:** ^1^Department of Physical Therapy, Thomas Jefferson University, Philadelphia, PA 19107, USA; ^2^Department of Pathology, Anatomy & Cell Biology (retired), Thomas Jefferson University, Philadelphia, PA 19107, USA; ^3^Department of Radiology, Thomas Jefferson University, Philadelphia, PA 19107, USA

## Abstract

Spondylosis deformans is a type of spinal claw osteophytosis which can be found on the anterolateral vertebral bodies of any region, and which consists of protrusions of intervertebral disc tissue covered by a bony shell. We report here a case of thoracic spondylosis deformans and multilevel instrumented fusion found during routine dissection of a cadaver. Theories of the etiology of this condition are reviewed in general, and with respect to this specific case and the potential interaction of the presenting comorbidities. The clinical implications of these osteophytes, including musculoskeletal and visceral sequelae, are also discussed.

## 1. Introduction

Spondylosis deformans (SD) is a type of spinal osteophytosis of uncertain etiology which contains elements of both an osteophyte and an intervertebral disk (IVD) herniation. These nodules arise from Sharpey's fibers at the rim of the anterolateral vertebral endplates and grow towards the adjacent vertebral body [1]. This bony migration ultimately forms a bridge between vertebral bodies that covers an IVD tissue bulge and can thereby result in the fusion of the involved segments [2–4]. The bony protrusions of SD can be considered a type of claw osteophyte according to Nathan's classification system [5, 6] and have not been extensively studied in humans, and then almost exclusively in the lumbar spine [7, 8]. The usage of the term “spondylosis deformans” is more common in the veterinary than the human literature, but a wide variation in the nomenclature used for the condition in both human and animal studies has created some confusion. For example, Morgan and Biery [4] noted more than 10 different terms used in the literature to refer to SD. Regardless of the terminology used, awareness of the diverse patient presentations that can be seen with this type of spinal osteophytosis is essential for clinicians treating patients with either musculoskeletal or visceral complaints.

## 2. Case Presentation

**Figure 1:**
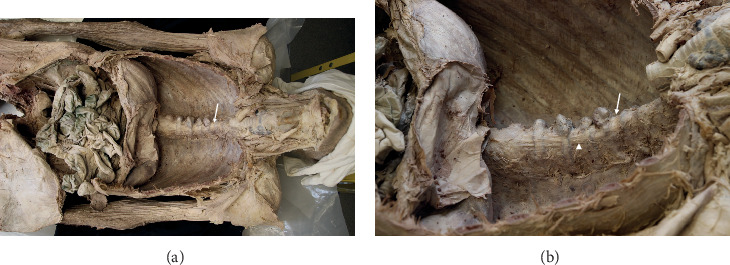
Thoracic osteophytes spanning vertebral bodies of T6 to T11. Arrows indicate T6 vertebral body, and the arrowhead the anterior longitudinal ligament. (a) Superior in situ view. (b) Left superolateral, close-up view.

Approval to conduct this research was received from the body donor program that provided our study subject, and exemption from human subjects review was obtained from the Thomas Jefferson University Office of Human Research. This report concerns observations made during a routine dissection of an 84-year-old male during a gross anatomy laboratory course. The cause of death was congestive heart failure and respiratory failure, with no other past medical history available. After removal of the anterior chest wall, the subject's bronchi, pulmonary vessels, and inferior vena cava were transected. The lungs were then removed and the heart reflected superiorly, revealing large osteophytes on the thoracic spine. These were noted to arise from the anterolateral vertebral bodies of T6 to T11 and to bridge the intervening IVDs (Figure 1).

**Figure 2:**
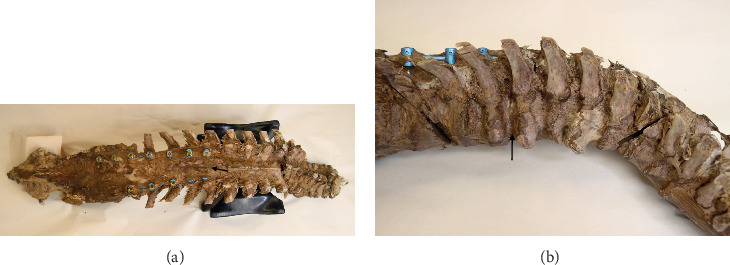
Disarticulated spine showing posterior fusion hardware and osteophytes. Arrows indicate T9 vertebral body. (a) Posterior view of entire spine. (b) Right, lateral, close-up view showing relationship of fusion hardware and osteophytes.

**Figure 3:**
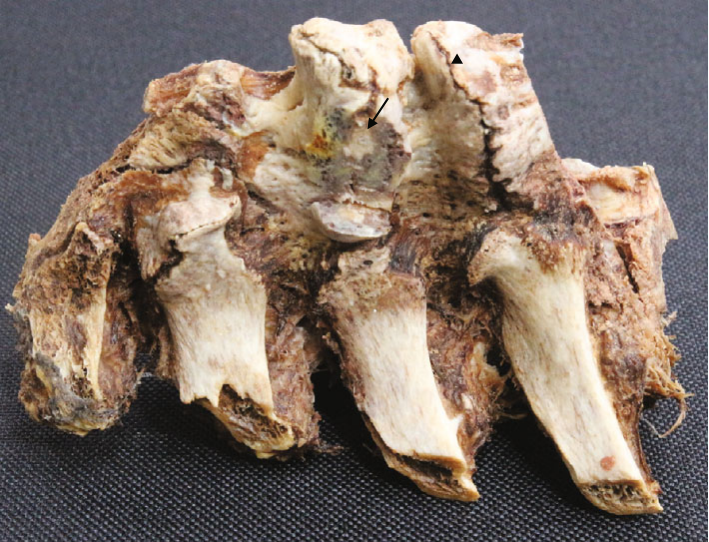
Section of disarticulated spine showing seam within osteophyte spanning T6-T7 IVD (arrowhead) and IVD tissue (arrow) contained within osteophyte spanning T7-T8 IVD.

The anterior longitudinal ligament was partially degraded where it contacted the osteophytes between T6 and T9 (Figure 1(b)). Although the esophagus remained oriented in the midline, its deep surface was noted to be in contact with osteophytes at T6-T7 and T8-T9. No compression of the sympathetic trunk by the osteophytes was found. At the conclusion of the anatomy lab course, the spine was disarticulated from the cadaver at the sacroiliac and atlantoaxial joint; at which time, hardware from an extensive thoracolumbar posterior fusion was noted. In order to expose the spine and hardware completely, residual soft tissue was removed by sharp dissection and successive immersion and rinsing using a bleach solution. This exposed the pedicle screws and rods spanning from T9 to L3 bilaterally, overlapping the thoracic osteophytes by two segments (Figure 2). A seam over the middle of the intervening IVD, a hallmark of SD, [8] showed clearly where the bony contributions from the involved vertebral bodies intersected (Figure 3). A wedge cut into one of the osteophytes was then made and IVD tissue was noted within the bone tissue surrounding the osteophyte (Figure 3).

## 3. Discussion

The pathology seen in the spine of our subject is complex and includes disk herniation, osteophytosis, multilevel instability, and both organic and surgical spinal fusion. Interactions among these conditions are very likely, but the lack of available medical history makes the sequence of onset of the various conditions unknown.

Although research on SD is very common in the veterinary literature [3, 4], human studies are few, and the specific etiology is unknown. Spondylosis deformans ultimately results in a type of organic spinal fusion, and thus it has been theorized that the unique osteophytes characteristic of SD form in order to correct an underlying spinal instability [4].

Physical stress, including obesity, is considered the primary cause of osteophyte formation [6, 9]. Biomechanical factors stimulate periosteal cells in the bone-cartilage interface to initiate the process of osteophyte formation, with evidence of a strong role for TGF beta and bone morphogenic protein in their formation [6]. Osteophyte incidence increases with age and may be associated with dietary and genetic factors [6], and spinal osteophytes arise in all regions of the spine, with the highest incidence in the lower thoracic region [5]. They can be subdivided into either traction subtypes, which curve away from the IVD or claw subtypes, which curve towards the IVD [6, 7]. Thoracic spine osteophytes are more frequently located on the right side, as seen with our subject, possibly due to interruption of spur formation on the left side caused by aortic pulsations [2].

Fissures in the annulus, a common age-related change in human and canine spines, have been noted to be a precursor to both SD and IVD herniation [4]. Anterior disk bulges have been associated with osteophyte formation, but the order of IVD disease versus osteophyte formation has not been well established [4, 10]. Anterior IVD herniation is less common than posterior herniation, likely due to human lifestyles involving repeated flexion, and also to morphological factors such as the thinner wall found in the posterior annulus [2]. As with anterior osteophytes, anterior disk herniations have been known to impinge upon visceral structures [11] and to cause visceral pain due to compression injuries of the sympathetic trunk [12].

Other common spinal lesions involving both the vertebral bodies and the IVDs can be differentiated from SD by considering the specific lesion location and anatomical features involved. For example, diffuse idiopathic skeletal hyperostosis (DISH) results in a fusion of spinal segments that is similar to the fusion seen with SD; however, DISH always involves ligament ossification [13]. In the spine, DISH creates a bridge over an intervening IVD and commonly impacts the anterior longitudinal ligament, which was largely spared in our subject. A Schmorl's node is another condition involving both the vertebral body and the IVD, but in this case, the IVD actually protrudes through the center of the cartilaginous endplate and into the cancellous bone at the center of the vertebral body [14].

The multilevel instrumented fusion seen in the present subject suggests that adjacent segment disease (ASD) was present in addition to the other pathologies previously discussed. Adjacent segment disease refers to pathological changes that arise in the mobile segments next to a spinal fusion. Spinal fusion surgeries are performed in cases of traumatic instability, or when decompression surgery at multiple levels creates this instability. The pathological manifestations of ASD can include degeneration, listhesis, instability, herniation, and osteophyte formation [15–17]. The etiologies of ASD involve complex biomechanical processes and can be categorized as leading to either increased degeneration or to increased motion in the neighboring segments. Older patients and those with multijoint involvement, 2 characteristics shared by our subject, are most at risk for ASD [15, 16, 18].

## 4. Conclusion

Our subject represents an unusual case of thoracic SD coupled with multilevel instrumented spinal fusion. The presence of SD in the thoracic spine is inherently contradictory because thoracic disk bulges are rare and represent fewer than 3% of all disk herniations [19], while thoracic osteophytes are quite common. The SD osteophytes seen in our subject may have developed, in part, secondary to ASD. Yet we have not found evidence in the literature that ASD can specifically cause the osteophytes characteristic of SD. Spondylosis deformans is itself a type of nonsurgical spinal fusion, and one study using a canine model showed that the organic fusion of SD can itself cause a type of ASD [20]. Regardless of the timing of onset of the conditions seen in the present case, awareness of thoracic SD and its potential complications is lacking among clinicians. This is important because while thoracic SD may be asymptomatic, it has the potential to cause dysphagia, respiratory issues, and compression of the sympathetic trunk or the aorta [6, 9, 21, 22]. There is a greater awareness among clinicians of the potential for cervical osteophytes to cause visceral symptoms, but imaging studies and diagnostic work-ups may miss thoracic involvement in, for example, dysphagia [22]. Particularly, when visceral symptoms occur in patients also experiencing back pain, clinicians should consider the possibility of the interaction of these two conditions. Awareness of the potential relationship between these bony deformities and visceral symptoms can prevent unnecessary testing and lead to appropriate referral for imaging, diagnostic work-up, and treatment.
